# Family planning practices and pregnancy intentions among HIV-positive and HIV-negative postpartum women in Swaziland: a cross sectional survey

**DOI:** 10.1186/1471-2393-13-150

**Published:** 2013-07-15

**Authors:** Charlotte E Warren, Timothy Abuya, Ian Askew

**Affiliations:** 1 Population Council, General Accident Insurance House, Ralph Bunche Road, P.O. Box 17643–00500, Nairobi, Kenya

**Keywords:** Pregnancy, Fertility desires, Postpartum care, Family planning, HIV

## Abstract

**Background:**

In settings where sexually transmitted infection (STI) and HIV prevalence is high, the postpartum period is a time of increased biological susceptibility to pregnancy related sepsis. Enabling women living with HIV to avoid unintended pregnancies during the postpartum period can reduce vertical transmission and maternal mortality associated with HIV infection. We describe family planning (FP) practices and fertility desires of HIV-positive and HIV-negative postpartum women in Swaziland.

**Methods:**

Data are drawn from a baseline survey of a four-year multi country prospective cohort study under the Integra Initiative, which is measuring the benefits and costs of providing integrated HIV and sexual and reproductive health (SRH) services in Kenya and Swaziland. We compare data from 386 HIV-positive women and 483 HIV-negative women recruited in Swaziland between February and August 2010. Data was collected on hand-held personal digital assistants (PDAs) covering fertility desires, mistimed or unwanted pregnancies and contraceptive use prior to their most recent pregnancy. Data were analysed using Stata 10.0. Descriptive statistics were conducted using the chi square test for categorical variables. Measures of effect were assessed using multivariate fixed effects logistic regression model accounting for clustering at facility level and the results are presented as adjusted odds ratios.

**Results:**

Majority (69.2%) of postpartum women reported that their most recent pregnancy was unintended with no differences between HIV-positive and HIV-negative women: OR: 0.96 (95% CI) (0.70, 1.32). Although, there were significant differences between HIV-positive and HIV-negative women who reported that their previous pregnancy was unwanted, (20.7% vs. 13.5%, p = 0.004), when adjusted this was not significant OR: 1.43 (0.92, 1.91). 47.2% of HIV-positive women said it was mistimed compared to 52.5%, OR: 0.79 (0.59, 1.06). 37.9% of all women said they do not want another child. Younger women were more likely to have unwanted pregnancies: OR: 1.12 (1.07, 1.12), while they were less likely to have mistimed births; OR: 0.82 (0.70, 0.97). Those with tertiary education were less likely to have unwanted or mistimed pregnancies OR: 0.30 (0.11, 0.86). Half of HIV-positive women and more than a third of HIV-negative women reported that they had been using a FP method when they became pregnant with no differences between the groups: OR: 1.61 (0.82,3.41). Only short-acting methods were available to these women before the most recent pregnancy; and available during the postpartum visit. One fifth of all women received an FP method during the current visit. Among the four fifths who did not receive a method 17.3% reported they were already using a method or were breastfeeding. HIV-positive women were more likely to have already started a method than HIV-negative women (20% vs. 15%, p = 0.089).

**Conclusion:**

There are few differences overall between the experiences of both HIV-positive and negative women in terms of FP experiences, unintended pregnancy and services received during the early postpartum period in Swaziland. Women attending postpartum facilities are receiving satisfactory care. Access to a wider range of effective methods is urgently needed if high levels of unintended pregnancy are to be reduced among HIV-positive and HIV-negative women living in Swaziland.

## Background

In virtually all Sub-Saharan African (SSA) countries, women have limited access to and use of health care services during the postnatal period [1]. A lack of clearly defined guidelines and standards in many countries, including the content and timing of postnatal care (PNC) for the mother and the baby up to six weeks after birth, contributes to a discontinuity with the services received during pregnancy and delivery [2,3]. In addition, postnatal guidelines do not cover women delivered by caesarean section, low-birth-weight or preterm babies, twins, new mothers and babies with certain health problems, adolescents and women living with human immunodeficiency virus (HIV) [3]. These gaps can limit linkages to other key services for new mothers, including family planning (FP) and HIV care services for women living with HIV [4].

HIV infection has become an important contributing cause of maternal mortality in Africa [5,6]. In settings where the prevalence of sexually transmitted infections (STIs), and HIV, is high, postnatal period is also a time of increased biological susceptibility to pregnancy-related sepsis [7] and the leading cause of maternal mortality. Women living with HIV are at 1.5 to 2 times greater risk of dying during pregnancy or childbirth than HIV-negative women [5,8] and are more likely to suffer from complications such as postpartum haemorrhage, puerperal sepsis and complications of caesarean section [9,10].

Globally, almost 90 million women have an unintended pregnancy each year, largely due to an unmet need for FP [11]. Providing FP to women in developing countries who have an unmet need for modern methods would prevent 54 million unintended pregnancies, including 21 million unplanned births, 26 million induced abortions (of which 16 million would be unsafe) and seven million miscarriages; this would also prevent 79,000 maternal deaths and 1.1 million infant deaths [11]. In SSA, the unintended pregnancy rate is estimated to be 20–40%, but only 21% of partnered women are using modern contraception and an estimated 20–35% of women have an unmet need for contraception [12]. Women are vulnerable to unintended pregnancy during the three to six months after delivery when they either reduce or stop exclusive breastfeeding and their natural fertility returns [13]. It estimated that 73% of women within one year after birth (the “extended postnatal period”) have an unmet need for FP [13].

Enabling women living with HIV to avoid unintended pregnancies during the postpartum period can reduce vertical transmission of HIV and maternal mortality associated with HIV infection [14]. For this reason, a key component of the World Health Organization’s (WHO) comprehensive strategy 2011–2015 for prevention of mother-to-child transmission (PMTCT) is to increase contraceptive use among HIV-positive women who wish to use it [15]. More recently there is emerging evidence of access to and use of FP by women living with HIV [16-19]. Of importance is evidence suggesting that in settings of low contraceptive prevalence, and high HIV prevalence, women living with HIV in most parts of SSA may have shorter birth spacing intervals than HIV-negative women [20,21], implying limited access to FP services following childbirth. However, there is limited evidence on fertility desires, contraceptive needs and FP practices of HIV-positive women during the postnatal period. This paper addresses these gaps by comparing fertility desires, family planning practices and receipt of PNC services among HIV-positive and HIV-negative post partum women in Swaziland.

## Methods

Data for these analyses are drawn from a baseline study of a four-year multi-country study - the Integra Initiative: which is measuring the benefits and costs of providing integrated HIV and sexual and reproductive health services in Kenya and Swaziland [22]. The study methodology and intervention is described in detail elsewhere [23]. Respondents were recruited between February and August 2010 as part of a prospective cohort study designed to measure the effect of timing and content of an integrated HIV and PNC/FP services model. This model developed explicit linkages with FP services and relevant HIV/AIDS services, for the mother and her baby. The intervention focussed on strengthening existing postpartum consultations during pre-discharge, one week, and six-week, additional consultations were introduced at six months to enable women to access time-relevant services for themselves and their babies. Moreover, information about and encouragement to receive this full package of postpartum services was made during antenatal-care consultations to increase continuum of care of essential services. The services included repeat HIV testing for mother, HIV testing for infant and referral to HIV services for HIV positive mothers and infants, as well as referrals for clients requiring additional services.

To assess the impact of service integration, the cohort of women were recruited from health facilities where they had attended for postnatal services and followed over a two year period. However this paper compares the fertility desires, family planning practices, information and services received during postnatal visits including breastfeeding, family planning counselling and uptake among HIV-positive and HIV-negative women using only the cross sectional baseline data.

Ten facilities were purposively selected, based on a minimum number of postpartum women attending per month (to be able to achieve the necessary sample sizes) and the availability of HIV, PMTCT, postpartum, FP and immunisation services at these facilities. Samples of women who were at least 18 years old, lived in the facility’s catchment area, had given birth within the previous 0–10 weeks and were receiving PNC for themselves and/or their babies were recruited for interview irrespective of their HIV status. All women attending on the days of data collection were approached for interview consecutively until the requisite sample size was reached. The desired sample size of 989 was calculated to test the larger study hypothesis that exposure to the PNC model of intervention would lead to an increase in condom use by at least 7 percent among sexually active women over two years.

A total of 886 women reported that they had been tested for their HIV status. Of these, 503 women reported being HIV-negative and 344 reported being HIV-positive; 29 women did not want to disclose their HIV status and 9 had tested but had not received their results. In addition to using self-reported status of HIV, we sought to validate these reports by examining responses to other questions to identify the services that the women had received during their previous antenatal or current postnatal visits. This process indicated that 42 women who self-reported as being HIV-negative had received HIV related services, suggesting that they were HIV-positive. This paper compares data from the subset of 386 women self-reporting as HIV-positive or assumed to be HIV-positive because of their use of HIV services, with the subset of 483 women self-reporting as HIV-negative and who had not used any HIV services. For the combined sample size of 869 women, the proportion of 44% considered to be HIV-positive mirrors the national HIV prevalence rate.

Each eligible respondent, willing to be interviewed, gave their informed consent prior to being interviewed. Teams of trained research assistants conducted the interviews using hand-held personal digital assistants (PDAs) loaded with the questionnaire translated from English into siSwati. The closed-ended questions on fertility desires focussed on the number of children born, whether the woman would like to have another child or not, their desired number of children and when they would like to have their next child. Mistimed or unwanted pregnancies were determined by asking whether, during the last pregnancy, the respondent wanted to be pregnant then, wanted to wait until later or did not want any more children.

Women were asked whether they were using any form of contraceptive method prior to their most recent pregnancy and if so which one(s). In addition, they were asked whether they had received any methods during the current visit, their preferred methods and the provider’s actions around FP counselling and service delivery. Women were also asked about their use of postpartum and postnatal services and previous use of STI/HIV services, including their knowledge of STI/HIV counselling and testing services and whether the provider offered counselling and testing for HIV during the current visit, whether the women accepted the test and if not why. Subsequently they were asked if they had been tested before and whether they had received the test results and were willing to disclose their status. The interviewers reiterated that providing this information was entirely optional and their response would be kept strictly confidential as no names or other identifiers were recorded on the data collection instrument; respondents were told that not disclosing their status was not a criterion for exclusion from the study and would not affect their ability to access services at the facility.

### Statistical analysis

Data recorded on the PDAs were imported into Microsoft Access and then into Stata 10.0 for analysis. All statistical tests were two-tailed, and interpreted at a 5% confidence level. Two methods of analysis were used. First, FP practices and service use by HIV-positive women was compared according to the time when they learnt their status in order to determine whether knowledge of being HIV-positive was an influence. Secondly, service use by all women was compared by the women’s HIV status. In both approaches, descriptive statistics were conducted using the chi square test for categorical variables; Fisher’s exact test was used for small cell sizes (<5) and a *T*-test was used to compare means across two groups.

Measures of effect were assessed using multivariate fixed effects logistic regression model accounting for clustering at facility level and the results were presented as adjusted odds ratios or incidence rate ratios (IRR). The basic model is given by Equation (1) where π_*ij*_ is the probability of experiencing the outcome for individual *i* identified from facility *j* ; *X*_*ij*_ is the vector of covariates; *β* is the associated vector of fixed parameters; and *μ*_*j*_ are the unobserved characteristics of individual identified from the same facilities.

(1) $$\log\mathrm{it}\left(\pi_{\mathrm{ij}}\right)=X_{\mathrm{ij}}\beta +\mu_{j}$$

The key outcome variables were previous fertility preferences (unwanted or mistimed births), use of FP when previous pregnancies was unwanted, future fertility intentions, and receipt of FP during current visit. The independent variable of interest was HIV status and was dichotomized into two categories (1 = HIV-positive and 0 = HIV-negative). The model controlled for education, marital status, age and whether they knew their HIV status before or after index pregnancy.

### Ethical issues

Researchers were trained on conduct of ethical procedures and monitored during fieldwork. We obtained informed consent for each study participant. All participants were given detailed information about the study including: aims, methods of study; institutional affiliations of the research; anticipated benefits, risks/discomfort and follow-up of the study; the length of the interview; the choice of not answering any questions and the right to abstain from participating in the study, or to withdraw from it at any time, without reprisal; measures were taken to ensure confidentiality and anonymity of information provided; the conduct of interviews in places of the participant’s choosing to maximize audio privacy; contact details of the study coordinator for any questions or concerns.

The study was approved by the Scientific Ethics Committee of the Swaziland Ministry of Health (MOH) (approval number MH/599C), the Ethics Review Committee of the London School of Hygiene & Tropical Medicine (LSHTM) (approval number 5426) and the Population Council institutional review board (IRB approval number 444). The Integra Initiative is registered on the Clinical Trials registration site: ClinicalTrials.gov Identifier: NCT01694862.

## Results

### Characteristics of women attending postnatal services

Table 1 describes the characteristics of women by HIV status whose ages ranged from 18 to 45 years and who attended for postnatal services for themselves or their infant on the day of interview. The parity among HIV-positive women was significantly higher than HIV-negative women when adjusted for age [p < 0.001]. This is also reflected by their age distribution with HIV-positive women being significantly older than HIV-negative women by two years [p < 0.001]. Desired family size was identical for HIV-positive and negative women, although there were differences between HIV-positive women in regard to when they found out their HIV status; women who knew their status before the most recent pregnancy desired 3.3 children compared to 2.5 for women finding out during the last pregnancy [p = 0.010].

**Table 1:** Socio-demographics profile of postpartum women

	**HIV-positive**	**HIV-negative**	**All women**	P value*
**Age (years)**	**386 (%)**	**483 (%)**	**869 (%)**	
18-25	175 (45.3)	315 (65.2)	490 (56.4)	
26-30	129 (33.4)	91 (18.8)	220 (25.3)	<0.001
31-35	61 (15.8)	41 (8.5)	102 (11.7)	
36-45	21 (5.4)	36 (7.5)	57 (6.6)	
**Pregnancies**				
Average number of pregnancies (SD)	2.9 (1.5)	2.4 (1.6)	2.6 (1.6)	<0.001
Average number of desired children (SD)	2.6 (1.4)	2.6 (1.2)	2.6 (1.3)	0.845
**Marital status**				
Single divorced	4 (1.0)	4 (0.8)	8 (0.9)	
Single in relationship	160 (41.5)	245 ( 50.7)	405 (46.6)	0.042
Single living with partner	47 (12.2)	42 (8.7)	89 (10.2)	
Married	175 (45.3)	192 (39.8)	367 (42.2)	
**Education**				
None	25 (6.5)	16 (3.3)	41 (4.7)	
Primary	126 (32.6)	115 (23.8)	241 (27.7)	0.001
Secondary	224 (58.0)	327 (67.7)	551 (63.4)	
Tertiary	11 (2.8)	25 (5.2)	36 (4.1)	
**Religion**				
None	18 (4.7)	20 (4.1)	38 (4.4)	
Christian	325 (84.2)	408 (84.5)	733 (84.3)	0.929
Traditional	43 (11.1)	55 (11.4)	98 (11.3)	
**When started antenatal care**	**362**	**432**	**794**	
1 to 3 months	87 (24.0)	67 (15.5)	154 (19.4)	0.002
4 to 6 months	248 (68.5)	325 (75.2)	573 (72.2)	0.035
7 to 9 months	27 (7.5)	40 (9.3)	67 (8.4)	0.363
**Place of index pregnancy delivery**	**386**	**483**	**869**	
Health facility	331 (85.8)	424 (87.8)	720 (86.8)	
Home	46 (11.9)	54 (11.2)	95 (11.5)	0.407
TBA/Relative	2 (0.5)	2 (0.4)	4 (0.5)	
On the way	7 (1.8)	3 (0.6)	9 (1.2)	

Across all tables*p values compares HIV + and HIV-negative women.

Almost all women (99.4%) had attended ANC services for the most recent pregnancy. However, HIV-positive women were significantly more likely to have attended for ANC services during the first trimester (1–3 months) of pregnancy [p = 0.002], especially those who knew their status before this pregnancy. Similarly high proportions (about 86%) of HIV-positive and HIV-negative women had given birth in a health facility.

### Fertility preferences

Over two thirds of the women interviewed reported that their pregnancy had been unintended, that is, either unwanted or mistimed i.e. occurred earlier than desired, with similar proportions among HIV-positive and HIV-negative women with no significant differences between the two groups (Table 2).

**Table 2:** Fertility preferences and pre-pregnancy use of FP methods among postpartum women

	**HIV-positive**	**HIV-negative**	**Total**	P value*
**Previous fertility preferences**	**386** (%)	**483** (%)	**869** (%)	
Unwanted or mistimed births	262 (67.9)	339 (70.2)	601 (69.2)	0.464
- Unwanted	80 (20.7)	65 (13.5)	145 (16.7)	0.004
- Mistimed	182 (47.2)	274 (56.7)	456 (52.5)	0.005
**Used FP when last pregnancy was**	**80**	**65**	**145**	
Unwanted	50 (62.5)	31 (47.7)	81 (55.9)	0.074
FP method used when woman became pregnant and did not want pregnancy**	**50**	**31**	**81**	
Hormonal pills and condoms	1 (2.0)	0 (0.0)	1 (1.2)	0.428
Hormonal pills only	4 (8.0)	10 (32.3)	14 (17.3)	0.005
Injectables and condoms	1 (2.0)	1 (3.2)	2 (2.5)	0.730
Injectables only	13 (26.0)	9 (29.0)	22 (27.2)	0.766
Implant only	0 (0.0)	1 (3.2)	1 (1.2)	0.201
IUCD only	0 (0.0)	1 (3.2)	1 (1.2)	0.201
Male condoms only	34 (68.0)	10 (32.3)	44 (54.3)	0.003
Withdrawal only	0 (0.0)	1 (3.2)	1 (1.2)	0.201
**Used FP when last pregnancy was**	**182**	**274**	**601**	
Mistimed	82 (45.1)	101 (36.9)	183 (30.4)	0.08
**FP used when woman became pregnant with mistimed pregnancy**	**82**	**101**	**183**	
Hormonal pills only	14 (17.1)	21 (20.8)	35 (19.1)	0.525
Injectable and condoms	2 (2.4)	0 (0.0)	2 (1.1)	0.115
Injectables only	29 (35.4)	35 (34.7)	64 (34.9)	0.920
Male condoms only	42 (51.2)	42 (41.5)	84 (45.9)	0.268
Emergency pills	0 (0.0)	1 (1.0)	1 (0.6)	0.366
Withdrawal and condoms	2 (2.4)	3 (2.9)	5 (2.7)	0.266
**Future fertility intentions**	**386**	**483**	**869**	
Does not want another child	154 (39.9)	175 (36.2)	329 (37.9)	0.268
**Length of time to next child**	**15**	**42**	**57**	
Wants another child within two to three years	1 (6.7)	2 (4.8)	3 (5.3)	0.777
Wants another child after three years	14 (93.3)	40 (95.2)	54 (94.7)	0.777

*** Multiple responses for types of methods used when pregnant.

There were significant differences between HIV-positive and HIV-negative women regarding the nature of their unintended pregnancy (Table 2), although more women living with HIV reported that it had been unwanted, when adjusted, the differences were not significant (Table 3). Fewer HIV-positive women reported a mistimed pregnancy [p = 0.005]. However, when adjusted for clustering and other variables, the likelihood of a mistimed birth reduces with increasing age: OR: 0.94, 95% CI (0.95, 0.96), [p = 0.009]. Women who were single and in relationship were to times likely to have a mistimed birth: OR: 2.05 (1.43, 2.81); [p < 0.001].

**Table 3:** Relationship between pregnancy intentions, pre-pregnancy use of FP methods and socio-demographics

	**Unwanted pregnancy**	**Mistimed birth**	**Unwanted or mistimed birth**	**Use of FP when last pregnancy was unwanted**	**Use of FP when last pregnancy was mistimed**
HIV status (HIV positive = 1)	1.43 (0.92, 1.91)	0.79 (0.59, 1.06)	0.96 (0.70.1.32)	1.61 (0.82,3.41)	1.32 (0.91,2.02)
Age (range 18–45 years)	1.12** (1.07,1.12)	0.82** (0.70,0.97)	1.23* (1.02,1.47)	1.16* (1.04,1.12)	1.02 (0.91,1.13)
Period of knowing HIV status (before index pregnancy =1)	1.34 (0.72,2.81)	0.81 (0.66,1.00)	0.86 (0.69,1.04)	0.57 (0.13,1.72)	0.65 (0.21,1.72)
**Education**					
Primary level	0.95 (0.42,1.90)	0.84 (0.42,1.70)	0.64 (0.28,1.46)	2.42 (0.6,10.7)	0.83 (0.26,2.17)
Secondary level	0.65 (0.34,1.32)	0.91 (0.46,1.77)	0.58 (0.26,1.30)	1.94 (0.56,8.11)	1.23 (0.52,3.21)
Tertiary	0.23 (0.04,1.04)	0.69 (0.27, 1.79)	0.30* (0.11,.0.86)	NA	1.92 (0.23,2.13)
**Marital status**					
Single divorced	13.23* (2.67,71.12)	0.45 (0.08,2.35)	5.22 (0.62,43.3)	0.87 (0.12,5.91)	NA
Single in relationship	1.76* (1.12, 2.71)	2.06** (1.51,2.82)	3.51** (2.47, 4.98)	1.73 (0.72,4.31)	0.61 (0.31.0.91)
Single living with partner	1.23 (0.61, 2.41)	1.53 (0.94,2.50)	1.69* (1.02.2.82)	0.94 (0.23,3.81)	1.08 (0.51,2.06)

*p < 0.05; **p < 0.01.

Younger women were more likely to have unwanted pregnancy; OR: 1.12, (1.07, 1.12); [p = 0.042], this was also the case for single or divorced women and single women in relationship: OR; 13.23 (2.67, 71.12), [p = 0.003] and OR; (1.76 (1.12, 2.76), [p = 0.001] respectively. Women who were either single and in a relationship or single living with a partner were three or two times likely to have an unintended pregnancy; OR: 3.51, (2.47, 4.98); [p < 0.001] and OR: 1.76, (1.12, 2.71), [p = 0.034]. Women with tertiary education were less likely to have an unwanted or mistimed births OR: 0.33 (0.11, 0.86), [p < 0.001] (Table 3).

One third of all women who had indicated an unwanted pregnancy reported that they had been using a FP method when they became pregnant; moreover, this proportion was not significantly different both at descriptive and when adjusted: 62.5% among HIV-positive and 47.7% among HIV-negative women [p = 0.074]. The majority of these women had used a short term method (condom, hormonal pill or injectable). For those reporting an unwanted pregnancy, HIV-positive women were more likely than HIV-negative women to be using male condoms and less likely to be using hormonal pills. More than half [59.2%] of all women who indicated a mistimed pregnancy reported they were using a short term FP method when they became pregnant with 65.1% of HIV-positive women stating this compared to 36.9% of HIV-negative women. However there were no significant differences by HIV status on type of FP method used. Over a third of all women stated they did not want another child with no differences by HIV status.

### Information and services for maternal and child health during postnatal visit

Over 40% of all women with no differences by HIV status reported receiving information from health service providers on the importance of waiting for at least two years before thinking about another pregnancy during the current visit. But fewer reported receiving information on when to expect return of menses, return of fertility or advice on when to commence sexual activity following childbirth (Table 4). There were no significant differences in information received by HIV status. The majority of HIV-positive women (89.4%) said they received information on infant feeding and were more likely than HIV-negative women (84.5%) to receive this information, especially those who knew their HIV status prior to the most recent pregnancy (95.1%). Less than a fifth of all women interviewed received any information on which danger signs they should look for in the newborn or very young infant. Examples of these signs include difficulty breathing, difficulty feeding, high or low temperature, jaundice and abnormal crying. There were no significant differences between HIV-positive and HIV-negative women for these indicators.

**Table 4:** Postpartum and postnatal service use among postpartum women by HIV status

**Providers gave information on:**	**HIV-positive**	**HIV-negative**	**Total**	P value*
	**213 (%)**	**304 (%)**	**517 (%)**	
Waiting before getting pregnant	92 (43.2)	127 (41.8)	219 (42.4)	0.748
	**383**	**482**	**865**	
Return to menstruation	71 (18.4)	81 (16.8)	152 (17.5)	0.506
	**386**	**483**	**869**	
Return to sexual activity	52 (13.4)	54 (11.8)	106 (12.2)	0.305
Infant feeding practices	345 (89.4)	408 (84.5)	753 (86.7)	0.035
	**385**	**478**	**863**	
Return to fertility	75 (19.5)	79 (16.5)	154 (17.8)	0.260
	**386**	**479**	**862**	
Danger signs in babies	59 (15.4)	92 (19.2)	151 (17.5)	0.145
	**360**	**429**	**789**	
FP after birth	273 (75.8)	281 (65.5)	554 (70.2)	0.002
**Infant feeding practices**	**386**	**483**	**869**	
Exclusive breast feeding	339 (87.8)	451 (93.4)	790 (90.9)	<0.001
Replacement feeding	46 (11.9)	8 (1.7)	54 (6.2)	
Mixed feeding	1 (0.3)	24 (6.2)	25 (2.9)	
Resumed sexual activity	78(20.2)	91(18.8)	169(19.5)	0.613
**% sexually active**	**78**	**91**	**169**	
Breastfeeding and not using FP ***	49 (62.8)	62 (68.1)	111 (65.6)	0.468
Breastfeeding and using a FP method	24 (30.7)	24 (26.4)	48 (28.4)	0.528
Not breastfeeding and not using FP	4 (5.1)	1 (1.1)	5 (2.9)	0.123
**Percent of women who**	**386**	**483**	**869**	
Received FP method during visit	69 (17.9)	97 (20.1)	166 (19.1)	0.411
**Methods received during current visit†**	**69**	**97**	**166**	
Hormonal pills only	10 (14.5)	29 (29.9)	39 (23.5)	0.021
Injectables only	45 (65.2)	58(59.8)	103 (62.0)	0.478
Male condoms only	11(15.9)	6 (6.1)	17 (10.2)	0.041
Female condoms	5 (7.2)	3 (3.1)	8 (4.8)	0.218
Condoms with another method	11 (15.9)	8 (8.3)	19 (11.5)	0.125
Intra uterine device	1 (1.5)	0 (0.0)	1 (0.6)	0.234
Female sterilization	0 (0.0)	1 (1.0)	1 (0.6)	0.398
Implants only	0(0.0)	1 (1.0)	1 (0.6)	0.398
**Reasons for not receiving method**	**316**	**385**	**701**	
Already using FP	63 (19.9)	58 (15.1)	121 (17.3)	0.089
Not ready for a method	166 (52.5)	213 (55.3)	379 (54.1)	0460
Health system factors	16 (5.1)	34 (8.8)	50 (7.1)	0.054
Personal factors	9 (2.8)	7 (1.8)	16 (2.3)	0.364
Others	62 (19.6)	73 (19.0)	135 (15.5)	0.826

*** Breastfeeding cases include only those that are exclusively breastfeeding while FP users is derived from those already in a method and those who received a method during the current visit.

† Multiple responses.

### Breastfeeding and use of FP

The vast majority of recently delivered women (90.9%) said they were exclusively breastfeeding their infants after childbirth, with significantly higher proportions of HIV-negative women (93.4%) than HIV-positive women (87.8%) (Table 3). However, HIV-positive women who knew their status before this pregnancy were more likely to breastfeed their babies and less likely to give replacement feeds than women who discovered they were HIV-positive during their pregnancy.

Among these women, 20.2% of HIV-positive and 18.8% of HIV-negative women had resumed sex since childbirth (Table 4), with approximately eight percent of all women having had sex within the first four weeks, with no difference by HIV status (data not in table). Two thirds of sexually active postpartum women were exclusively breastfeeding (and not using FP) less than one third were both breastfeeding and using FP. Among those not breastfeeding, five women were using FP; another five were neither using FP nor breastfeeding and so theoretically at risk of an unintended pregnancy because of an unmet need for contraception.

### Family planning counselling and uptake

The majority of all women reported having discussed FP with the provider during their visit; this proportion was significantly higher for HIV-positive women (76%) than for HIV-negative women (66%). Approximately one-fifth of women reported receiving a FP method during the current visit; there were no significant differences between HIV-positive and HIV-negative women. The most frequently received method was the hormonal injectable (62.0%), followed by the hormonal pill (23.5%), and male or female condoms (11.5%); three women received a long-acting or permanent method. Significantly more HIV-positive than HIV-negative women received condoms and significantly fewer received contraceptive pills. Although not significant, more HIV-positive women were using a condom with another contraceptive method compared to HIV-negative women (15.9% versus 8.3%).

Among the four-fifths of women who did not receive a method during their postnatal consultation, 17.3% reported that they were already using a short term method. HIV-positive (20%) women were more likely to have already started using a method than HIV-negative women (15%), although this difference was not statistically significant. Most women (54%) not already using a method or not starting a method reported that they did not feel ready to start at this point in time. Seven percent of women reported wanting to start a method but had experienced a health system related barrier, such as their choice of method not being available, a lack of supplies or equipment, the provider being “too busy”, or being referred elsewhere for FP, and so are considered to have an unmet need.

## Discussion

Our findings show that HIV-positive women were on average older, more likely to have a higher parity and less education than HIV-negative women, an observation consistent with findings from other studies in SSA [20,21,24]. Two thirds of this sample of recently delivered women in Swaziland reported that their most recent pregnancy was unintended, a high level even for a country of southern Africa where high levels of unintended pregnancy occur [6]. A higher proportion of women reported that the pregnancy was mistimed rather than unwanted; the proportion reporting that the pregnancy had been unwanted was significantly higher among HIV-positive than HIV-negative women. As most HIV-positive women had already achieved their desired family size, this finding is not surprising but reflects a substantial unmet need for effective contraception for women wanting no more children.

However, more than half of all postpartum women said that they had been using a short term FP method when they became pregnant, and this proportion was significantly higher (around three fifths) among HIV-positive women. Moreover, two-thirds of HIV-positive women experiencing an unwanted pregnancy and half of those experiencing a mistimed pregnancy were using the condom. Reliance on condoms for dual protection to prevent both unintended pregnancy and HIV transmission or re-infection does not, therefore, appear to be an effective strategy, a finding supported by other studies [25].

Use of FP method prior to the previous pregnancy and the FP method available to the women on the day of interview are similar. The majority of methods available to this group of women are short term methods: hormonal pills, injectables and condoms even though a sizable number report not wanting any more children. Among women reporting that the last pregnancy was unwanted, it is not known whether their use of a short-acting, less effective method was because of preference for such methods or because of the limited availability of longer-acting, more effective methods. Methods such as the implant, intra-uterine device and sterilization are not readily available in Swaziland’s public health facilities [26], indeed, hormonal implants were only introduced in late 2010 (personal communication 2011 with Head of Sexual and Reproductive Health Unit ,Ministry of Health). The intra-uterine device is only available if there is a skilled provider within the facility (often only one individual) and sterilization through referral to a higher level facility.

Service providers frequently miss opportunities to counsel all women, including those living with HIV, on the full range of contraceptive methods [20]. Some studies have demonstrated that long acting methods are not necessarily recommended by providers or accessible to women living with HIV due to limited knowledge of the health care workers providing HIV services knowledge and counselling skills; lack of commodities operational guidelines and poorly integrated reproductive health/FP and HIV services [27]. Nevertheless, a study in Rwanda demonstrated an increase in use of implants among HIV-positive women (who had recently given birth) when access was improved [28,29], suggesting that this intervention is both feasible and acceptable.

Although a substantial proportion of women received information from health care providers on delaying their next pregnancy for at least two years and on FP, few received information on when to expect return to fertility and menses. At the time of interview many reported using a contraceptive method prior to the postnatal visit, received an FP method in the current visit or reported exclusively breastfeeding and so do not, according to some definitions, have an unmet need for FP. However if women are not clear when their fertility is likely to return these women will be potentially at risk in a few months time.

A few women had resumed sexual activity following childbirth but were not exclusively breastfeeding and so natural fertility is likely to return soon. In addition a proportion of non breastfeeding women were also not using any contraception. The likelihood of becoming pregnant again in the next three to six months is high as found in other studies [13]. This demonstrates that women receive information on FP but are only receiving short term methods even though majority do not want any more children.

One key limitation of this study is the fact that the study population of postpartum women attending a health facility is not representative of the general postpartum population in Swaziland, as only 25% of newly delivered women attend for PNC [26]. However the findings do reflect the services received by recently delivered women in most public health facilities in Swaziland. Another limitation is that HIV status was self-reported by the interviewees and validated through referencing other questions on use of HIV services and not through the maternal card or through health facility records. The proportion of women determined to be HIV-positive (44.4%) reflects the HIV prevalence among pregnant women in the country, which was 42 percent in 2008 [26], thus suggesting that this measure is probably valid.

## Conclusion

There are few differences overall between the experiences of both HIV-positive and HIV-negative women in terms of use of FP experiences, of unintended pregnancy and services received during the postpartum period. However, key differences do exist. HIV-positive women appear more likely to have an unwanted pregnancy and less likely to have a mistimed pregnancy than HIV-negative women. HIV-positive women were also more likely to have used condoms prior to the unwanted pregnancy. The women were in the first few weeks after delivery, and therefore due to the high proportion of women who are breastfeeding and not sexually active indicates that these women are indeed protected against another pregnancy immediately. This indicates that women in the period following childbirth in Swaziland are receiving satisfactory care. However, the main weakness is the lack of access or availability of long acting and permanent methods, given the high proportion of women having unintended pregnancies and not wanting any more children.

## Competing interests

The authors declare that they have no competing interests.

## Authors’ contributions

CW as involved in the design of the project, conceptualising the study and drafting the initial draft. TA was involved in analysis of the data, drafting the manuscript and revising all the changes. IA was involved in the project design, conceptualising the study and review the manuscript for intellectual content. All authors read and approved the final manuscript.

## Pre-publication history

The pre-publication history for this paper can be accessed here:

http://www.biomedcentral.com/1471-2393/13/150/prepub
